# Pathological Voice Source Analysis System Using a Flow Waveform-Matched Biomechanical Model

**DOI:** 10.1155/2018/3158439

**Published:** 2018-07-02

**Authors:** Xiaojun Zhang, Lingling Gu, Wei Wei, Di Wu, Zhi Tao, Heming Zhao

**Affiliations:** ^1^School of Electronic and Information Engineering, Soochow University, Suzhou 215000, China; ^2^College of Physics, Optoelectronics and Energy, Soochow University, Suzhou 215000, China

## Abstract

Voice production occurs through vocal cord and vibration coupled to glottal airflow. Vocal cord lesions affect the vocal system and lead to voice disorders. In this paper, a pathological voice source analysis system is designed. This study integrates nonlinear dynamics with an optimized asymmetric two-mass model to explore nonlinear characteristics of vocal cord vibration, and changes in acoustic parameters, such as fundamental frequency, caused by distinct subglottal pressure and varying degrees of vocal cord paralysis are analyzed. Various samples of sustained vowel /a/ of normal and pathological voices were extracted from MEEI (Massachusetts Eye and Ear Infirmary) database. A fitting procedure combining genetic particle swarm optimization and a quasi-Newton method was developed to optimize the biomechanical model parameters and match the targeted voice source. Experimental results validate the applicability of the proposed model to reproduce vocal cord vibration with high accuracy, and show that paralyzed vocal cord increases the model coupling stiffness.

## 1. Introduction

Vocal cord vibration interrupts the straight airflow expelled by the lungs into a series of pulses that act as the excitation source for voice and sound. Denervation or organic diseases of vocal cords, such as paralysis and polyps, can cause irregular vibration with consequential changes, manifested as breathy or hoarse voice. These diseases generally affect one side of vocal structure, causing significant imbalance in bilateral vocal cord tension [1, 2]. Irregular vibration of the vocal cords corresponding to a variety of voice disorders can be observed with electronic laryngoscope to assist diagnosing vocal cord disease. However, laryngoscopy examination is invasive, and the outcomes are relatively subjective. Acoustic analysis can complement and in some cases replace the other invasive methods, which based on direct vocal fold observation [3, 4].

Clinical diagnosis and pathological voice classification using objective methods is an important issue in medical evaluation. Previous studies have mainly combined acoustic parameters with pattern recognition algorithms to assist diagnosis of pathological voice [5, 6]. However, the selected voice signal parameters are not directly linked with the actual physical structure, and vocal structural changes that cause vocal voice disorders require further study.

Nonlinear dynamics theory has provided a new avenue for dynamical system related research, for example, methods combining nonlinear theory with spectral analysis have been successfully applied to EEG and ECG signal analysis. It has also been extended to study voice signals [7, 8].

Nonlinearity inherent in the vocal system can cause irregular voice behavior, as indicated by harmonics, bifurcation, and low-dimensional chaos in high-speed recording of vocal cord vibration signals [9, 10]. The degree of pathological vocal fold is closely related to the nonlinear vibration of the vocal cords. [11]. Therefore, traditional analysis of acoustic parameters may not be accurate, but nonlinear dynamics theory has been shown to have good applicability in characterizing such signals [12]. Time frequency shape analysis based on embedding phase space plots and nonlinear dynamics methods can be used to evaluate the vocal fold dynamics during phonation [13]. Nonlinear models can also simulate various vocal sound phenomena and have been used for dynamic prediction of disordered speech associated with larynx pathology [14–16]. Many physical modeling methods for glottal excitation have been proposed, and the corresponding model parameters have been utilized to study various voice disorders. The two-mass (IF) model is the most well-known classical physical model of the vocal cords, first proposed by Ishizaka and Flanagan and simplified by Steinecke and Herzel (SH model), to study vibration characteristics of the vocal cords. Xue combined the work of Steinecke and Herzel with Navier-Stokes equations and analyzed irregular vibrations caused by tension imbalance in bilateral vocal cord, as well as sound effects [17]. Recently, Sommer modified the asymmetric vocal contact force of the SH model based on Newton's third law [18]. However, a comprehensive nonlinear analysis for the modified SH model remains incomplete.

Although physical modeling has enormous potential in speech synthesis and voice analysis, the large number of model parameters and the complexity of model optimization to match observational data have prevented its practical application [19]. Döllinger used the Nelder–Mead algorithm to minimize the error between experimental curves obtained from high-speed glottography sequences and curves generated with the two-mass model (2MM) [20]. However, this is an invasive method because an endoscope is required to record vocal cord vibrations during phonation. Gómez computed biomechanical parameters based on the power spectral density of the glottal source to improve detection of voice pathology [21].

Other researchers have used genetic algorithms to optimize model parameters to match recorded glottal area, trajectory, and glottal volume wave and have shown the possibility of model inversion [22, 23]. Tao extracted the physiologically relevant parameters of the vocal fold model from high-speed video image series [24].

The complex optimization process and large number of parameters mean the matching result can be unstable. Thus, finding the important tuning parameters and selecting appropriate optimization algorithms are still important issues to be resolved for physical modeling applications, and simulations for asymmetric vocal cords also require further study.

This paper designed a pathological voice source analysis system using an optimized model to study the dynamics of asymmetric vocal cords. Incorporating spectral analysis, and bifurcation and phase diagrams, this paper investigates the impact of structural change of the vocal cord on its vibration and fundamental frequency. Sound effects due to lung pressure are also studied. An optimized SH model combined with particle swarm and quasi-Newton methods (GPSO-QN) is proposed to determine biomechanical model parameters. Parameter adjustments and changing the oscillation mode of the model allow normal and paralyzed voice sources to be simulated. Differences between optimized model parameters are analyzed to assist in identifying the source of vocal paralysis.

## 2. Method

### 2.1. Symmetric Vocal Model

**Figure 1:**
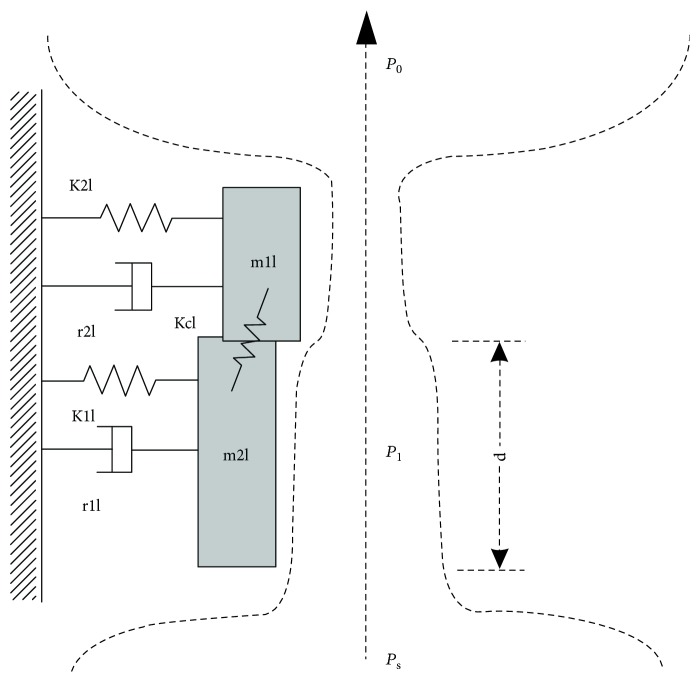
Schematic of the Herzel and Steinecke model.

Vocal cords are two symmetrical membranous anatomical structures located in the throat. Airflow out of the trachea and lungs continuously impacts the vocal cords and causes vibration. The vibration behavior modulates the airflow to generate glottal pulses [25]. Based on the elastic and dynamic properties of the vocal cords, each fold is represented by two coupled oscillators with two masses, three springs, and two dampers, where the quality of the mass and spring constants denote vocal quality and tension, respectively.  Figure 1 shows the simplified two-mass (SH) model, which can be expressed as

(1) $$\begin{array}{c} \overset{.}{x_{1\alpha }}=\upsilon_{1\alpha },\\ {\overset{.}{\upsilon }}_{1\alpha }=-\frac{1}{m_{1\alpha }}\left(F_{1\alpha }+I_{1\alpha }-r_{1\alpha }\upsilon_{1\alpha }-k_{1\alpha }x_{1\alpha }-k_{c\alpha }\left(x_{1\alpha }-x_{2\alpha }\right)\right),\\ \overset{.}{x_{2\alpha }}=\upsilon_{2\alpha },\\ {\overset{.}{\upsilon }}_{2\alpha }=-\frac{1}{m_{2\alpha }}\left(I_{2\alpha }-r_{2\alpha }\upsilon_{2\alpha }-k_{2\alpha }x_{2\alpha }-k_{2\alpha }\left(x_{2\alpha }-x_{1\alpha }\right)\right), \end{array}$$

where

(2) $$\begin{array}{c} F_{1\alpha }=\frac{{LdP}_{1}}{m_{1\alpha }},\\ I_{i\alpha }=-\Theta \left(-a_{i}\right)\frac{c_{i\alpha }}{m_{i\alpha }}\frac{a_{i}}{2L},\\ \Theta \left(x\right)=\left\{\begin{array}{c} 1,x>0\\ 0,x<0, \end{array}\right.\\ a_{i}=a_{0i}+L\left(x_{il}+x_{ir}\right),\\ a_{\min}=\min\left(a_{1},a_{2}\right), \end{array}$$

index *i* = 1, 2 denotes the upper and lower mass, respectively; *α* = *l*, *r* denotes the left and right parts, respectively; *P*_*s*_ is the subglottal pressure; *x*_*iα*_ and *v*_*iα*_ are the displacement and corresponding velocity of the masses, respectively; *m*_*iα*_, *k*_*iα*_, *k*_*cα*_, and *r*_*iα*_ represent the mass, spring constant, coupling constant, and damping constant, respectively; *L*, *d*, and *a*_0*i*_ represent the vocal cord length, thickness of mass *m*_1*α*_, and rest area, respectively; *c*_*ia*_ = 3*k*_*ia*_ is an additional spring constant for handling collision; *a*_*i*_ is the glottal area; *F*_1*a*_ and *I*_*ia*_ are the Bernoulli force and restoring force due to vocal cord collision, respectively; and *P*_1_ is the pressure on the lower masses.

Using aerodynamic analysis, pressure drops at the glottal entrance and viscous loss within the glottis is ignored.

In contrast to the IF model, Bernoulli flow exists below the narrowest glottis gap only, with a jet region above the contraction where pressure is considered to be constant [26]. From Bernoulli's equation,

(3) $$\begin{array}{c} P_{s}=P_{1}+\frac{\rho }{2}{\left(\frac{U_{g}}{a_{1}}\right)}^{2}=P_{0}+\frac{\rho }{2}{\left(\frac{U_{g}}{a_{\min}}\right)}^{2}, \end{array}$$

where *P*_0_ is the supraglottal pressure, *U*_*g*_ is volume flow velocity (glottal waveform), and *ρ* is air density. We ignore channel coupling, that is, *P*_0_ = 0, and consider that Bernoulli pressure exists only when the glottis is open. Therefore,

(4) $$\begin{array}{c} P_{1}=P_{s}\left[1-\Omega \left(a_{\min}\right){\left(\frac{a_{\min}}{a_{1}}\right)}^{2}\right]\Omega \left(a_{1}\right), \end{array}$$

(5) $$\begin{array}{c} U_{g}=\sqrt{2P_{s}/\rho }a_{\min}\Theta \left(a_{\min}\right), \end{array}$$

where

(6) $$\begin{array}{c} \Omega \left(x\right)=\left\{\begin{array}{cc} \tanh\left[50\left(x/x_{0}\right)\right], & x>0\\ 0, & x<0, \end{array}\right. \end{array}$$

with the units centimeters, grams, and milliseconds, respectively.

**Figure 2:**
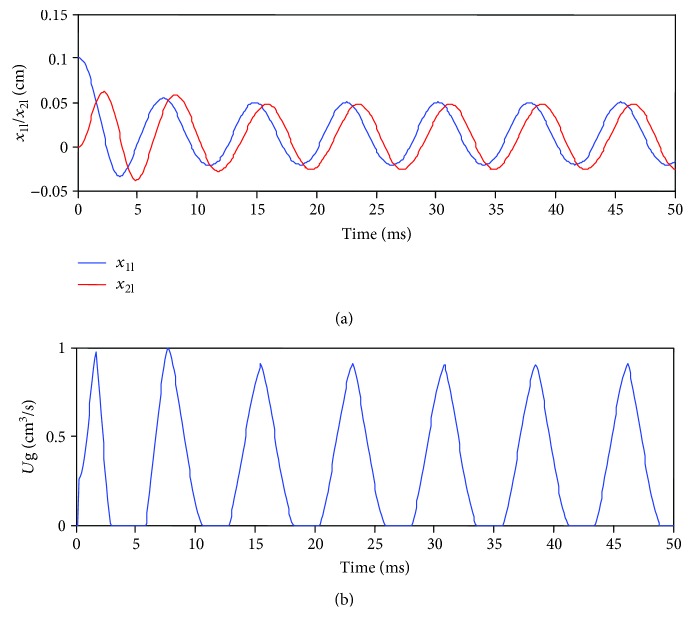
Simulation of the standard symmetric model showing oscillation of (a) left lower and upper masses (*x*_1l_ and *x*_2l_, resp.), and (b) glottal volume flow velocity *U*_*g*_.

The standard parameters of this model are *m*_1α_ = 0.125, *m*_2α_ = 0.025, *k*_1α_ = 0.08, *k*_2α_ = 0.008, *k*_cα_ = 0.025, *r*_1α_ = *r*_2α_ = 0.02, *P*s = 0.008, *d* = 0.25, *a*_01_ = *a*_02_ = 0.05, and *L* = 1.4. These parameters are used by the symmetric model to simulate vocal cord vibration, solving the differential equations using the standard fourth order Runge-Kutta method with initial conditions *x*_1α_(0) = 0.01, *x*_2α_(0) = 0.01, *v*_1α_(0) = 0, and *v*_2α_(0) = 0, as shown in Figure 2. Displacement of upper and lower masses and glottal airflow waveforms are cyclical, and a fixed phase difference exists for the displacement waveform (see Figure 2(a)).

### 2.2. Asymmetric Vocal Cord Model

Vocal polyps and paralysis often occur in one side of the vocal cords. Asymmetric vocal cords cause tension imbalance, and overcritical imbalance may cause irregular vibration. Without loss of generality, we assume the left vocal cord is normal, that is, unchanged parameters, and lesions occur only on the right vocal cord. This imbalance is represented by an asymmetry parameter *β*(0.4 < *β* ≤ 1), and right vocal parameters can be expressed as

(7) $$\begin{array}{c} {\tilde{m}}_{ir}=\frac{m_{ir}}{\beta },\\ {\tilde{k}}_{ir}=\beta k_{ir},\\ {\tilde{k}}_{cr}=\beta k_{cr},\\ {\tilde{c}}_{ir}=\beta c_{ir}. \end{array}$$

Small *β* means a high degree of asymmetry and leads to more complex vocal cord vibration. Consequently, subharmonic performance is enhanced and chaos occurs. Bifurcation diagrams and phase portraits can be used to describe the impact of *β* changes on the vocal system.

When the vocal cords are asymmetric, contact forces are modified as

(8) $$\begin{array}{c} I_{ir}=-\Theta \left(-a_{i}\right)\frac{c_{ir}}{m_{ir}}\frac{a_{i}}{L}\left(\frac{1}{\beta +1}\right),\\ I_{il}=-\Theta \left(-a_{i}\right)\frac{c_{il}}{m_{il}}\frac{a_{i}}{L}\left(\frac{\beta }{\beta +1}\right). \end{array}$$

### 2.3. Analysis of Vocal Vibration

Vibration characteristics of the asymmetric two-mass model were analyzed with respect to time, frequency, and phase. The vocal mechanism of clinical pathological voice was also investigated with respect to physical simulation. As discussed above, we assumed the left vocal cord was normal, and lesions occurred only in the right vocal cord. Clinical observation of vocal cord physiological characteristics suggested 0.4 < *β* ≤ 1 was an appropriate range and subglottal pressure was fixed at 0.8 kPa.

**Figure 3:**
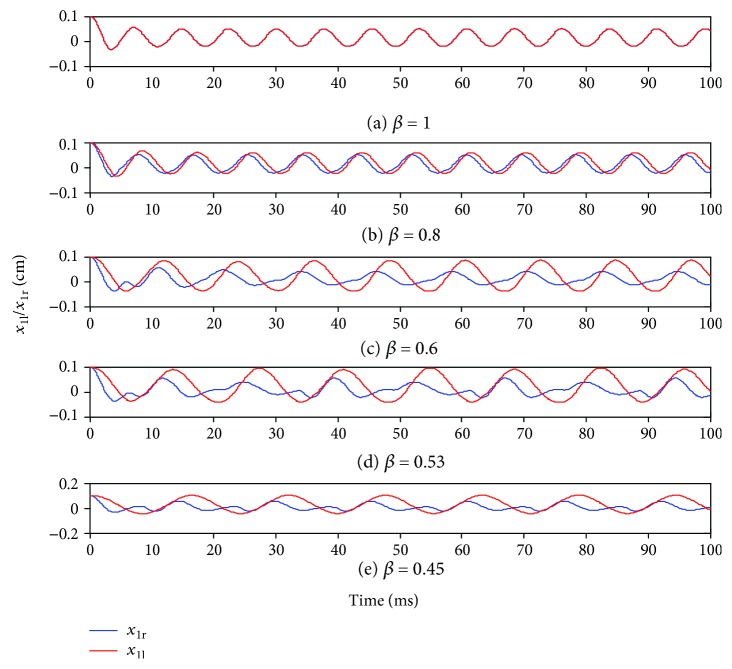
Mass displacements of the lower left and right sides.

Figure 3 shows displacement of the lower bilateral mass for *β* = 0.45, 0.53, 0.6, 0.8, and 1. Vocal cords on both sides were structurally symmetrical for normal voice, and the vibrational waveforms on both sides coincided completely. Duration of the vocal opening and closing once is defined as one pitch period, and there exists one maximum value of *x*_ir_ in such a period.

Asymmetric vocal cord vibrations are significantly more complex. When the degree of asymmetry was relatively small (*β* = 0.8), right vocal amplitude was slightly larger than the left side, and the phase was relatively advanced. As the degree of asymmetry increases, right vocal amplitude also increases with left amplitude remaining essentially unchanged. Consequently, phase difference increases, and the extrema ratio of both sides is no longer 1 : 1. Figure 3(d) shows the extrema ratio changes to 1 : 3, and quasiperiodic or irregular oscillations appear, leading to irregular airflow velocity.

**Figure 4:**
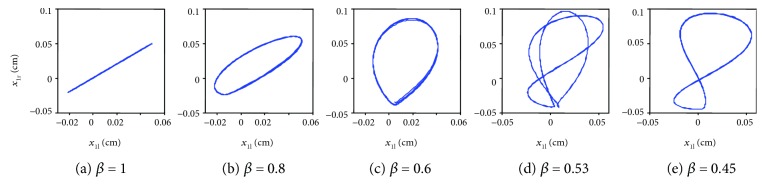
Phase space portrait in the *x*_1l_ − *x*_1r_ plane.

Before and after bifurcation, evolution of the dynamical systems in phase space can be described with phase diagrams of the displacement of bilateral vocal cord vibration in the *x*_1l_ − *x*_1r_ plane.  Figure 4 shows that when *β* = 0.8, no bifurcation occurs, and the phase trajectory is a limit cycle. As *β* reduces to 0.53, asymmetry increases, bifurcation appears, and the phase trajectory becomes a complicated period doubling limit cycle. However, when *β* = 0.45, the phase trajectory geometry simplifies, which is consistent with the results in the time domain.

**Figure 5:**
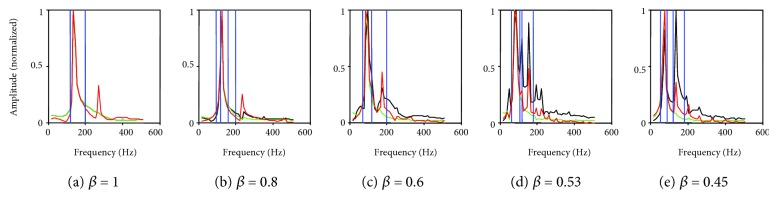
Fourier spectra corresponding to displacement of the two lower masses and the normalized glottal volume flow rate with *P*s = 0.8 kPa. Volume flow rate and left and right mass displacement are represented by the red, black, and green lines, respectively. Vertical dash lines represent the two left vocal cord natural frequencies, and the dash-dot lines represent those of the right vocal cord.

Considering the cases with fixed subglottal pressure (0.8 kPa) and *β* = 0.45, 0.53, 0.6, 0.8, and 1, we compared Fourier spectra corresponding to *x*_1l_, *x*_1r_, *U*_*g*_, and the natural frequencies obtained from an eigenvalue analysis of the system. Figures 5(a)–(e) show two vertical dashed lines that represent the two natural frequencies of the left vocal cord, and dash-dotted lines represent those of the right vocal cord.

When *β* = 1 (Figure 5(a)), the healthy phonation case and the bilateral folds have the same natural frequency. This phonation frequency is approximately 145 Hz, located between the two eigenfrequencies of the left (or right) side. As *β* reduces, the eigenfrequencies do not coincide again and more complex vibratory behaviors are observed. Figure 5(b) shows that for less asymmetry, *β* = 0.8, although the intrinsic frequency changes, there is relatively little effect on the frequency spectrum. Figure 5(c) shows that when *β* = 0.6, a frequency approximately 190 Hz with relatively small amplitude appears between the two eigenfrequencies of the left normal folds. Figure 5(d) shows that when *β* = 0.53, the overlapped frequencies of the preexisting overtone separate and a small overtone frequency appears between them at 110 Hz. Figure 5(e) shows that when *β* = 0.45, the overtone between the second eigenfrequency of the right fold and the first left fold disappears. However, the amplitude of the overtone frequency between the eigenfrequencies of the left normal folds becomes nearly as large as the pitch frequency.

Thus, the fundamental frequency is mainly dependent on the pathological vocal cords, while the normal folds mainly influence the overtone.

## 3. Model Parameter Optimization

We propose an optimization process to find appropriate parameters for the biomechanical model that can accurately simulate normal and paralyzed voice sources. First, inverse filtering is implemented to reduce the channel effect on the speech signal, and glottal flow is extracted. Glottal flow is separately parameterized in time and frequency domains to reduce computational complexity. Then, an optimization algorithm is employed to optimize SH model parameters to obtain a simulated glottal flow. Finally, minimizing error between the parameters of the simulated and extracted glottal flows allows the model to accurately reproduce the particular voice source, and corresponding vocal parameters can also be obtained.

### 3.1. Estimation of the Glottal Source

Reconstruction of the glottal source is based on the adaptive version of iterative inverse filtering developed by Alku [27]. The voice trace, *s*, may be considered as the output of a generation model, *f*_*g*_, excited by a train pulse, *δ*, whose output is modeled by the vocal tract transfer function, *f*_*v*_ to, yield voice at the lips, *s*_*l*_, which is radiated as *s*, where *r* is the radiation model, that is, ^∗^ means convolution of signals,

(9) $$\begin{array}{c} s=\left(\left(\delta *f_{g}\right)*f_{v}\right)*r=\left(f_{g}*f_{v}\right)\left.*r\right)=s_{l}*r \end{array}$$

**Figure 6:**
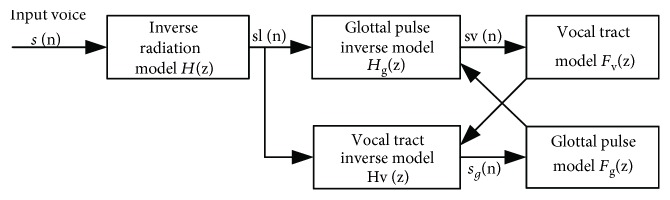
Estimation of the glottal pulse *s*_*g*_(n) by iterative filtering.

Figure 6 shows the inverse filtering procedure. The radiation effect is first removed by *H*(z), and the resulting radiation compensated voice, *s*_*l*_(n), is filtered by *H*_*g*_(z) to reconstruct the deglottalized voice, *s*_*v*_(n), from which the estimate of *F*_*v*_(z) may be derived. The vocal tract inverse model fed with the *F*_*v*_(z) filter parameters was used to remove the influence of the vocal tract from *s*_*l*_(n), producing a first estimate of the glottal pulse, *s*_*g*_(n). Another iteration was started with the new estimated *H*_*g*_(z) loaded by *F*_*g*_(z), and the cycle repeated 2 or 3 times to obtain a good estimation of the glottal source.

The glottal flow will be defined as

(10) $$\begin{array}{c} u_{g}\left(n\right)=s_{g}\left(n\right)‐{\bar{s}}_{g}\left(n\right). \end{array}$$

**Figure 7:**
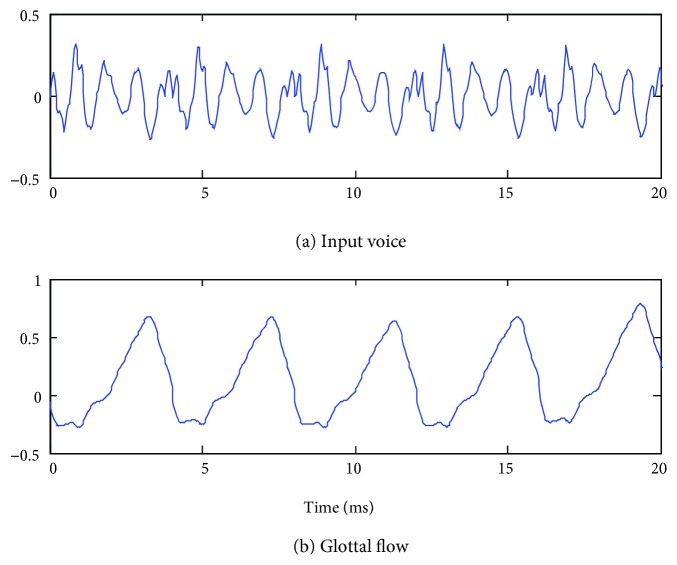
Example from vovel /a/ for a normal speaker.

An example of the glottal flow estimation from inverse filtering is shown in Figure 7.

### 3.2. Objective Function Vocal Cord

Since the asymmetric SH model influences oscillations in both time and frequency domains, the glottal flow, *u*_*g*_, and simulated waveforms, *Ug*, were also parameterized within those domains for comparison frequency, *F*_0_, and time quotients based on the Lijiencrants-Frant model were calculated, including speed quotient (*SQ*), the ratio of the glottal opening to closing time open quotient (*OQ*), the ratio of the open time to the fundamental period; closing quotient (*CIQ*), the closing time divided by the fundamental period; and normalized amplitude quotients (*NAQ*), the ratio of amplitude quotients (maximum amplitude divided by corresponding maximum negative peak of its first derivative) to the fundamental period.

To describe the error between normal target glottal flow and simulated waveforms, the objective function, *FY*, was defined as

(11) $$\begin{array}{c} FY=\omega_{1}\left(\frac{\;|\;OQ-{OQ}^{'}\;|\;}{OQ}+\frac{\;|\;SQ-{SQ}^{'}\;|\;}{SQ}+\frac{\;|\;CIQ-{CIQ}^{'}\;|\;}{CIQ}+\right.\left.\frac{\;|\;NAQ-{NAQ}^{'}\;|\;}{NAQ}\right)+\omega_{2}\frac{\;|\;F_{0}-{F_{0}}^{'}\;|\;}{F_{0}}, \end{array}$$

where “′” means the parameters are derived from the simulation waveform.

Traditional perturbation analyses have shown instability of pathological vocal sound. The resultant objective function is defined as:

(12) $$\begin{array}{c} {FY}_{p}=\omega_{3}FY+\omega_{4}\left(\frac{\;|\;J_{OQ}-{J_{OQ}}^{'}\;|\;}{J_{OQ}}+\frac{\;|\;J_{SQ}-{J_{SQ}}^{'}\;|\;}{J_{SQ}}+\right.\left.\frac{\;|\;J_{CIQ}-{J_{CIQ}}^{'}\;|\;}{J_{CIQ}}+\frac{\;|\;J_{NAQ}-{J_{NAQ}}^{'}\;|\;}{J_{NAQ}}+\frac{\;|\;J_{{{}_{F}}_{0}}-{J_{F0}}^{'}\;|\;}{J_{{{}_{F}}_{0}}}\right), \end{array}$$

where variables with superscript denote parameters of the simulated glottal flow.

If the time-based quotients are equally weighted, the effect of frequency and time parameters on *F* are the same, and their differential impact on *F*_*p*_ is equivalent to the original parameters, that is, *ω*_1_ = 0.125 and *ω*_2_ = *ω*_3_ = *ω*_4_ = 0.5. When *F* or *F*_*p*_ reaches a global minimum, the corresponding model can accurately reproduce the target glottal waveform.

### 3.3. Optimization Algorithm

Gradient techniques have proven to be inadequate, since the objective function is nonconvex and contains many local minima. The evolutionary algorithm has high robustness, and broad applicability for global optimization can deal with complex problems that traditional optimization algorithms cannot solve. Particle swarm optimization (PSO) and genetic algorithm (GA) are similar but have various strengths in dealing with different problems [28].

Therefore, we combined their advantages. PSO is an evolutionary computation technique based on swarm intelligence and is a community-based optimization tool. The PSO algorithm first initializes a group of random particles with random solutions and then all individuals and the best individuals of groups breed. The optimal solution is found through an iterative process. We added selection and crossover processes similar to GA into PSO, generating a GPSO algorithm.

In contrast, the quasi-Newton method is commonly used for solving nonlinear optimization problems, where the gradient of the objective function at each iteration step is obtained. An objective function can be constructed from the measured gradient to produce superlinear convergence. However, this method is somewhat sensitive to the initial point, and results are mostly local optima. Therefore, we combined the GPSO and quasi-Newton algorithm (GPSO-QN) to optimize the biomechanical model parameters to match the target voice sources.

The masses, spring constants, coupling coefficients, damping constants, and subglottal pressure all need optimization, which can be expressed as a vector *Φ* = [*m*_iα_, *k*_iα_, *k*_cα_, *r*_iα_, *P*_s_]. With optimized *Φ* the model should simulate *U*g in good agreement with *u*_*g*_.

Previous analysis has shown that asymmetric pathological vocal cords are the leading cause of irregular vibration. Consequently, we took *Φ* and *β* as matching parameters with the search interval [*m*_iα_, *k*_iα_, *k*_cα_, *r*_iα_] ∈ [0.001, 0.5], *β* ∈ [0.4, 1], and *P*_s_ ∈ [0.001, 0.05]. Then suitable matching parameters can be obtained using the proposed GPSO-QN algorithm to ensure the optimized model accurately reproduces the glottal waveform.

To avoid obtaining local minima in a nonconvex search space by direct application of the gradient method, the GPSO algorithm is first applied to provide a rough approximation, and then the QN method is applied to locally optimize the approximate solution, providing the globally optimal result.

**Figure 8:**
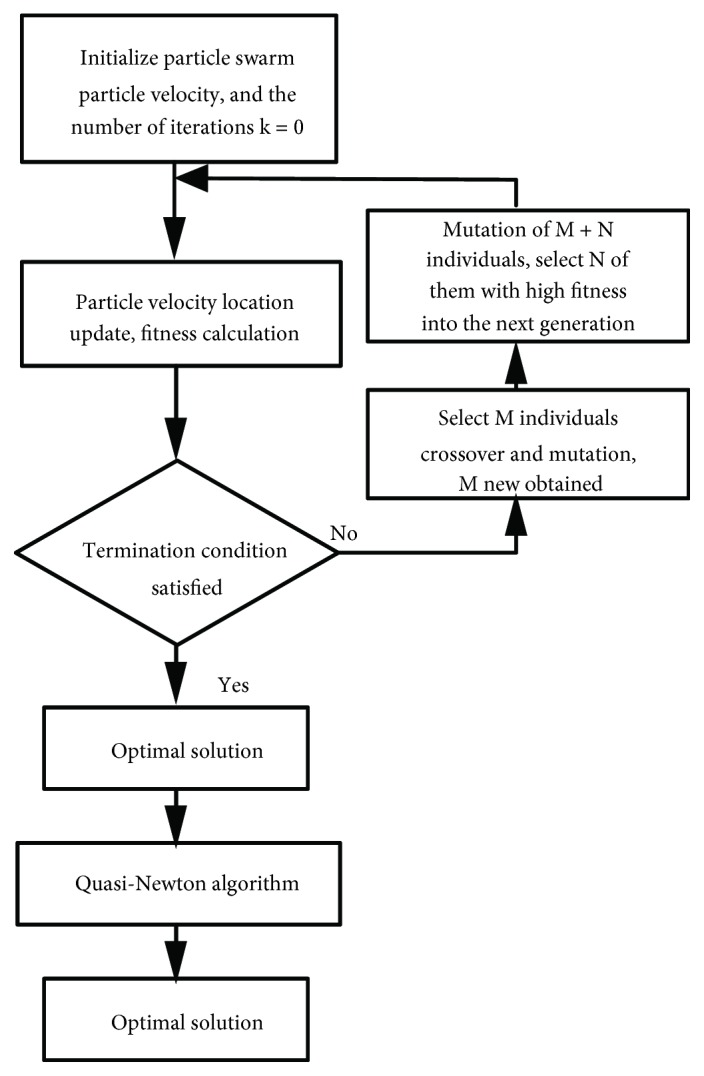
Proposed GPSO-QN algorithm structure.

Figure 8 shows the parameter optimization process. Selection and crossover process utilizes the Monte Carlo selection rule to choose *M* individuals. The termination condition is that the obtained maximum fitness value exceeds a preset threshold or the preset number of iterations is reached.

## 4. Result and Discussion

### 4.1. Experimental Parameters

This paper selected sustained vowel /a/ from the MEEI database [29], numbering the samples 1–8 (4 normal and 4 paralysis voices). Sampling frequency was 25 kHz, and the proposed GPSO-QN algorithm was used to optimize the model parameters with the number of particles for the initial population set as 30 and the number of generations limited to 400. Learning factors *c*_1_ and *c*_2_ were set = 2, and the range of weight coefficient *ω* was set = [0.5, 0.9].

### 4.2. Normal Voice Source Matching

**Figure 9:**
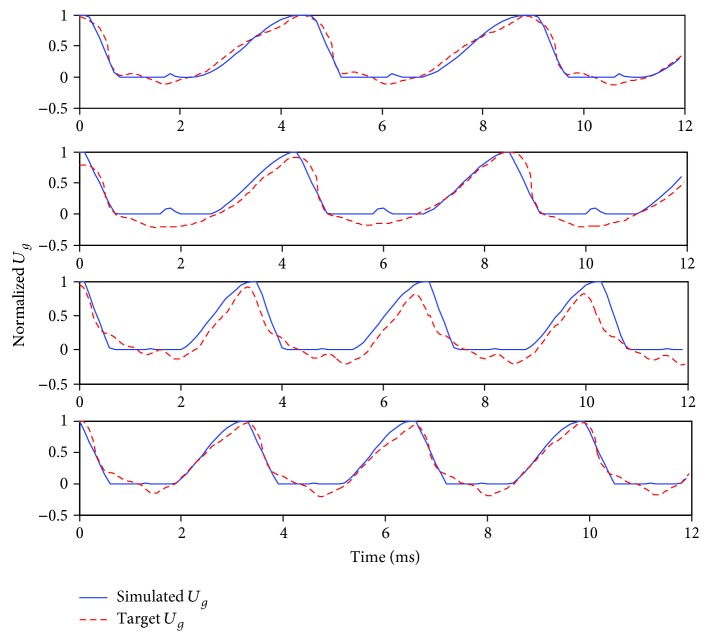
Matching result of normal voice source in the time domain.

**Figure 10:**
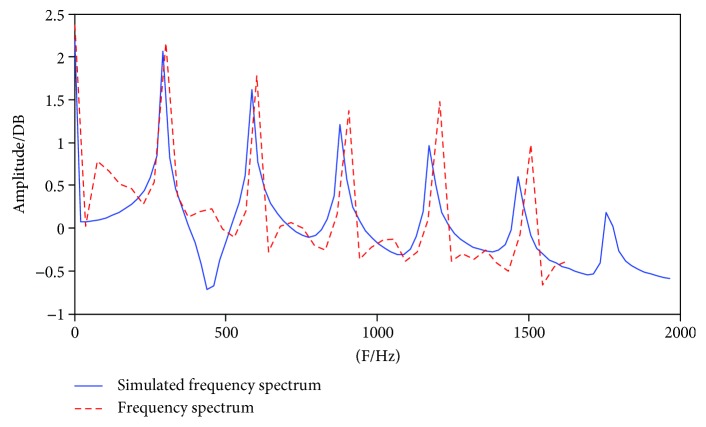
Matching result of sample 3 voice source in the frequency domain.

Figure 9 shows the excitation sources (red dashed lines) extracted from the four normal voice samples using the optimized model were accurately simulated. Using sample 3 as an example, Figure 10 shows that the simulated and actual spectra also have good consistency.

### 4.3. Paralysis Voice Source Matching

**Figure 11:**
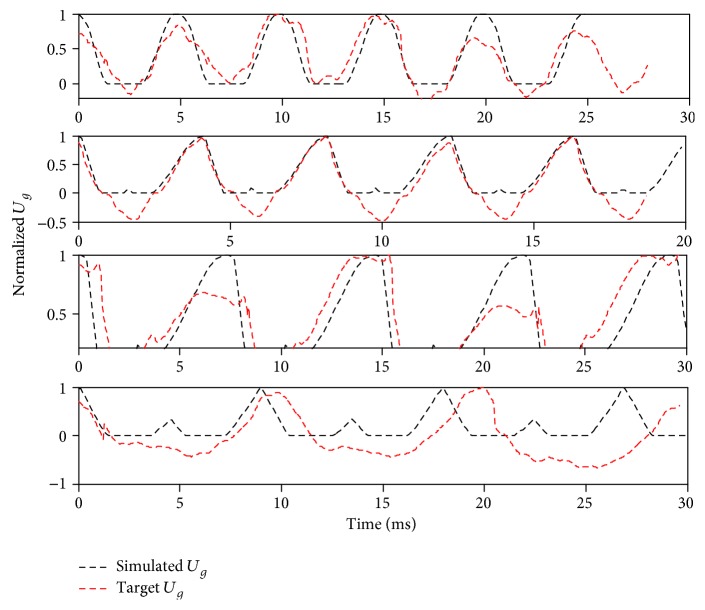
Matching result of paralysis voice source in the time domain.

**Figure 12:**
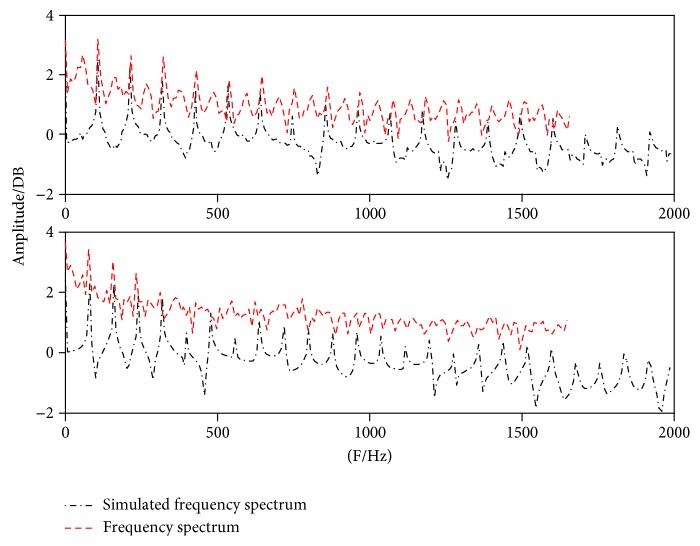
Matching results of samples 7 and 8 in the frequency domain.

Figure 11 shows that the model simulated waveforms for paralyzed voice samples (red dashed lines) have significant errors to actual samples, particularly for samples 7 and 8. However, the spectra show good consistency with only magnitude bias, as shown in Figure 12.

### 4.4. Difference Analysis of Matching Results

**Figure 13:**
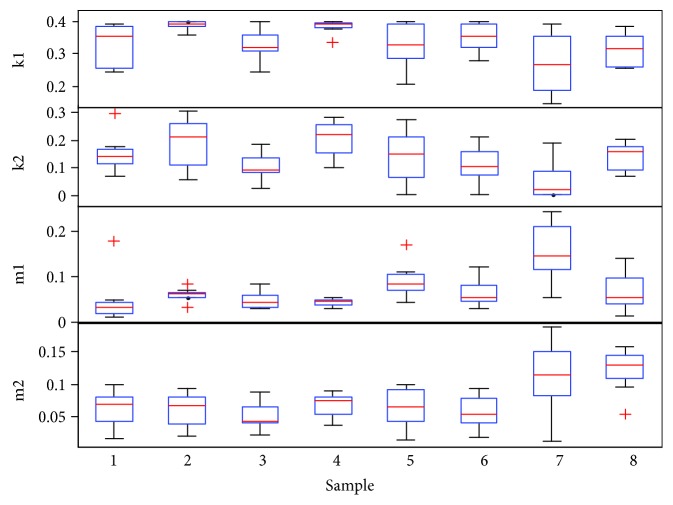
Statistical distribution of normal (1–4) and paralyzed samples (5–8).

To investigate the differences between normal and paralysis voice sources, we matched 9 consecutive frames of samples 1–8, and Figure 13 shows the statistical distribution of the optimized parameters. There were no significant differences between stiffness, quality, and damping of normal and paralysis models. However, the coupling stiffness of paralyzed vocal voice sources is greater than that of normal sources, and significant asymmetry in the paralyzed vocal cords was observed, as shown in the last two rows of Figure 13(b).

**Figure 14:**
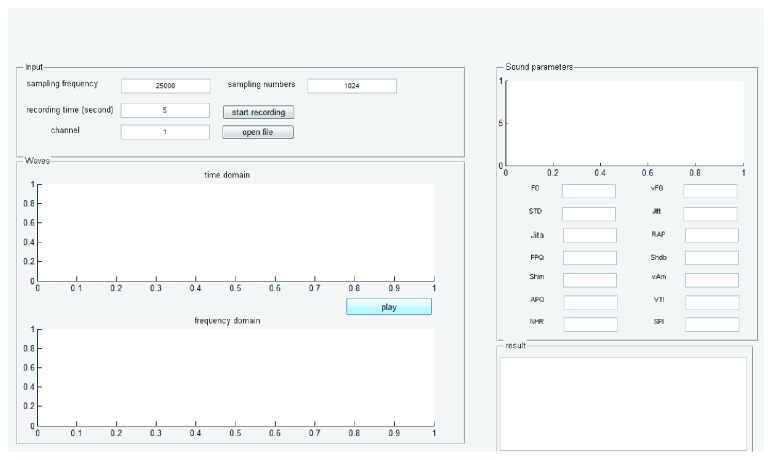
Pathological voice source analysis system.

Therefore, coupling stiffness and the asymmetry parameter, *β*, could be used as a basis for classifying normal and paralyzed vocal sources.  Figure 14 shows the pathological voice source analysis system. It is designed and programmed by MATLAB.

## 5. Conclusion

This study analyzed nonlinear characteristics of asymmetric vocal cord motion using an optimized biomechanical model to design a pathological voice source analysis system. A proposed algorithm was employed to optimize the masses, spring constants, coupling coefficient, damping constants, asymmetry parameter, and subglottal pressure of the mass model.

The proposed biomechanical model accurately simulated irregular vibration caused by unbalanced vocal tension. Period doubling bifurcation and frequency entrainment were observed in the bifurcation and phase diagrams, and spectrograms.

Vibration system complexity and asymmetry do not have a simple proportional relationship. This study shows that pitch frequency is mainly affected by the asymmetric structure of the vocal cord, whereas the impact of subglottal pressure is relatively small.

The optimal biomechanical model can accurately reproduce the voice source stream modulated by asymmetric vocal cords. Although the physiological parameters of voice sources were different, the asymmetry and coupling stiffness parameters helped determine paralysis voice sources.

Optimized model simulations will be of great value for understanding clinical hoarse voices corresponding to asymmetric vocal structure and predicting the effect on unilateral vocal disease treatment.

Future work will establish rational sound models for vocal cord polyps and other organic diseases to match real voice sources, assisting in classification of vocal cord diseases.

## Data Availability

The data used to support the findings of this study are available from the corresponding author upon request.
